# Malignant pericytes expressing GT198 give rise to tumor cells through angiogenesis

**DOI:** 10.18632/oncotarget.18196

**Published:** 2017-05-25

**Authors:** Liyong Zhang, Yan Wang, Mohammad H. Rashid, Min Liu, Kartik Angara, Nahid F. Mivechi, Nita J. Maihle, Ali S. Arbab, Lan Ko

**Affiliations:** ^1^ Georgia Cancer Center, Medical College of Georgia, Augusta University, Augusta, GA, USA; ^2^ Department of Molecular and Cellular Biology, Baylor College of Medicine, Houston, TX, USA; ^3^ Department of Biochemistry and Molecular Biology, Medical College of Georgia, Augusta University, Augusta, GA, USA; ^4^ Department of Pathology, Medical College of Georgia, Augusta University, Augusta, GA, USA

**Keywords:** angiogenesis, pericytes, oral cancer, glioblastoma

## Abstract

Angiogenesis promotes tumor development. Understanding the crucial factors regulating tumor angiogenesis may reveal new therapeutic targets. Human GT198 (*PSMC3IP* or Hop2) is an oncoprotein encoded by a DNA repair gene that is overexpressed in tumor stromal vasculature to stimulate the expression of angiogenic factors. Here we show that pericytes expressing GT198 give rise to tumor cells through angiogenesis. GT198^+^ pericytes and perivascular cells are commonly present in the stromal compartment of various human solid tumors and rodent xenograft tumor models. In human oral cancer, GT198^+^ pericytes proliferate into GT198^+^ tumor cells, which migrate into lymph nodes. Increased GT198 expression is associated with increased lymph node metastasis and decreased progression-free survival in oral cancer patients. In rat brain U-251 glioblastoma xenografts, GT198^+^ pericytes of human tumor origin encase endothelial cells of rat origin to form mosaic angiogenic blood vessels, and differentiate into pericyte-derived tumor cells. The net effect is continued production of glioblastoma tumor cells from malignant pericytes via angiogenesis. In addition, activation of GT198 induces the expression of VEGF and promotes tube formation in cultured U251 cells. Furthermore, vaccination using GT198 protein as an antigen in mouse xenograft of GL261 glioma delayed tumor growth and prolonged mouse survival. Together, these findings suggest that GT198-expressing malignant pericytes can give rise to tumor cells through angiogenesis, and serve as a potential source of cells for distant metastasis. Hence, the oncoprotein GT198 has the potential to be a new target in anti-angiogenic therapies in human cancer.

## INTRODUCTION

Angiogenesis has long been recognized as a hallmark of cancer [1, 2], and is essential for the development of many, if not all, human solid tumors [3–5]. Clinical anti-angiogenesis therapies such as bevacizumab treatment have shown only transient benefits in cancer patient survival [6]. The challenge remains to reconcile conflicting observations in angiogenesis research, and to identify additional angiogenic factors with better therapeutic potential.

Angiogenesis with the formation of new blood vessels is a normal biological process during embryonic development and in adult tissue repair [7–9]. This process is hijacked in tumor development. In normal angiogenesis, under stimulation by angiogenic factors, endothelial cells first sprout from existing capillaries to provide a monolayer luminal structure. Pericytes are then recruited to enclose and stabilize these endothelial cells to form microvessels [8, 10]. Further remodeling and maturation of microvessels is facilitated by a complex molecular signaling network that establishes a stable new vasculature [7, 11]. Tumor angiogenesis appears to mirror normal angiogenesis with the exception of having defects characterized by continued microvessel formation and constant reconstruction of the vasculature [10]. Several interconnected hypotheses of tumor angiogenesis have been proposed [12–15]. These include: sprouting angiogenesis [16], the sprouting of endothelial cells from existing capillaries; vessel co-option [5], the adoption of existing vessels by tumor cells; intussusception [17], the splitting of existing vessels to form new ones; vascular mimicry [18–20], *de novo* formation of new vessels by tumor cells; vasculogenesis [21], recruiting bone-marrow derived progenitors to form new vessels; and the theory of cancer stem cells [22–24], the differentiation of tumor-derived stem cells into new vessels, and importantly, into pericytes [25, 26]. In this study, we provide evidence to support existing angiogenesis hypotheses [27], and suggest that the presence of oncoprotein-stimulated malignant pericytes is a shared defect in tumor angiogenesis.

Glioblastoma xenografts closely mimic primary human glioblastoma, a common and aggressive type of brain tumor with poor prognosis [28]. The U-251 glioblastoma xenograft is characterized by extensive angiogenesis with hypertrophic vascular proliferation [29]. Despite genetic instability [30], this glioblastoma cell line is an excellent model for studies on angiogenesis, in part because of the presence of CD133^+^ stem cells which transdifferentiate into vascular lineages [22, 24, 31]. The plastic and angiogenic nature of tumor stem/progenitors is crucial for tumor development since these cells can recapitulate new tumor vasculature. The mouse GL261 glioma also possesses similar characteristics to human glioblastoma, and has been extensively investigated as a mouse tumor model for testing immunotherapy [32, 33]. Although angiogenic stimuli or risk factors in human cancers versus rodent models may be distinct, the fundamental principles of tumor angiogenesis are likely shared.

The hypotheses regarding tumor angiogenesis are also in accordance with the theory of epithelial-to-mesenchymal transition (EMT), where mesenchymal stromal stem cells have been shown to give rise to angiogenic malignant pericytes [26]. Epithelial-to-mesenchymal transition is a process by which tumor cells become stromal stem cells, which can then differentiate into a variety of cell types and gain mobility and invasiveness during metastasis [34]. When differentiate into pericytes, they stimulate tumor angiogenesis by forming tumor vasculature with poor pericyte coverage. Clinical data have previously shown that low pericyte coverage in vasculature is correlated with decreased patient survival [35, 36]. These malignant pericytes may further acquire properties promoting their mobility and invasiveness during tumor metastasis. Thus, malignant pericytes may be of central importance for both tumor angiogenesis and tumor metastasis.

Here, we investigate malignant pericytes using the oncoprotein GT198 as a marker. The human *GT198* gene (*PSMC3IP*; encodes GT198, also known as Hop2 or TBPIP) is a DNA repair gene and also a breast and ovarian cancer gene located within the *BRCA1* locus at chromosome 17q21. Germline mutations in *GT198* have been identified in familial breast and ovarian cancer [37, 38]. Somatic mutations in *GT198* are prevalent in tumor stroma in sporadic breast, ovarian, and fallopian tube cancers [39–41]. *GT198* mutations induce a truncated active mutant form containing the GT198 DNA-binding domain, resulting in cytoplasmic GT198 expression [39, 40]. Tumor-specific cytoplasmic expression of GT198 has previously been demonstrated in angiogenic pericytes located within the mutant breast tumor stroma [41]. GT198 is a transcriptional coactivator stimulating a variety of target genes including adipogenic and angiogenic factors such as VEGF [41, 42]. GT198 directly stimulates the human VEGF promoter [41]. In addition, extensive analyses have shown that GT198 stimulates DNA strand exchange and regulates DNA recombination in both DNA repair and meiosis [43–46]. While the broad functions of GT198 in transcription and DNA repair may rely on its DNA-binding capacity [39, 43, 45], GT198 is also expressed in embryoid body stem cells [39], as well as in pericyte progenitors [41]. It is therefore conceivable that both transcription and DNA repair may be affected in stem/progenitors producing malignant pericytes, thereby impacting tumor angiogenesis and in turn cancer.

We have previously shown that cytoplasmic GT198 is expressed in mutant breast tumor stromal cells including in angiogenic pericytes [41]. In this study, we show that GT198^+^ pericytes are a common feature among various human solid tumors as well as rodent xenograft tumor models. Specifically, we show that GT198^+^ pericytes have malignant potential and give rise to tumor cells in both human oral cancer and in rat glioblastoma xenografts. We further show that GT198 vaccination in mouse glioma containing GT198^+^ pericytes resulted in suppressed tumor growth and prolonged mouse survival. Consistent with the notion that tumor progenitors actually evolve into pericytes [25, 26], we propose that malignant pericytes also can differentiate into tumor cells through the process of angiogenesis. The presence of GT198^+^ pericytes provides a rationale for the integration of existing hypotheses in tumor angiogenesis, and implies the potential of oncoprotein GT198 as a new target in anti-angiogenesis therapy.

## RESULTS

### GT198 expression in pericytes is a common feature in tumor stroma of human solid tumors and xenograft mouse tumors

**Figure 1:**
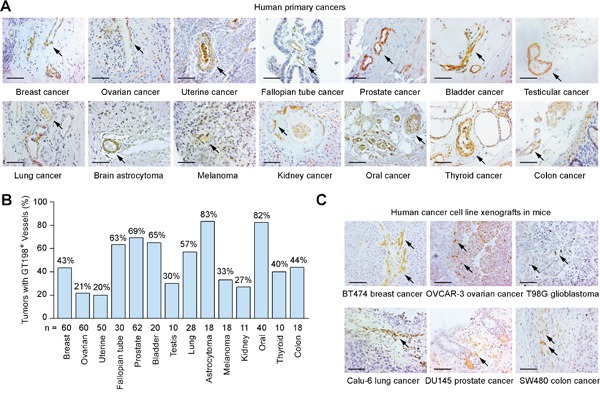
Pericytes expressing GT198 are common in human primary cancers and xenograft mouse tumors **(A)** Immunohistochemical staining of cytoplasmic GT198 in pericytes and perivascular cells in tumor stroma of human primary cancers. **(B)** Positive rates of GT198^+^ vessels in 14 types of human cancers. Number of cases (n) analyzed are indicated. **(C)** Immunohistochemical staining of GT198 in xenograft mouse tumors. Human cancer cell lines for implantation are indicated below. Arrows indicate GT198^+^ cells. Sections are counter-stained with hematoxylin. Scale bars = 100 μm.

Angiogenic pericytes expressing cytoplasmic GT198 have been previously identified in mutant breast cancer stroma [41]. Here, we further analyzed multiple human solid tumors for GT198^+^ pericytes by immunohistochemistry. GT198^+^ pericytes in capillaries, and perivascular cells in small vessels, were found in angiogenic tumor stroma of human primary cancers in breast, ovary, uterus, fallopian tube, prostate, bladder, testis, lung, brain, melanoma, kidney, oral cavity, thyroid, and colon (Figure 1A). Positive blood vessels were often not sporadically located but clustered in angiogenic tumor stroma where GT198^+^ fibroblasts were also present. In contrast, GT198^-^ pericytes in vessels were often located in non-angiogenic areas of tumor stroma (Supplementary Figure 1). The highest positive rates were found in brain astrocytoma (83%) and oral cancer (82%), which were mostly angiogenic (Figure 1B). Although *GT198* mutations have not been analyzed in every types of cancer, *GT198* somatic mutations that cause cytoplasmic GT198 overexpression have been previously identified in breast, ovarian, uterus, and fallopian tube cancer [37, 39–41]. The current results suggest that angiogenic GT198^+^ pericytes are frequently present in tumor stroma of multiple types of human solid tumors.

In addition, we analyzed xenograft mouse tumors produced by six human cancer cell lines including breast cancer BT474, ovarian cancer OVCAR-3, glioblastoma T98G, lung cancer Calu-6, prostate cancer DU145, and colon cancer SW480. We found GT198^+^ capillaries were present in all cases, particularly in angiogenic tumor stroma adjacent to tumor (Figure 1C). Thus, GT198 expression in pericytes is also a common feature in mouse transplantation tumor models.

### Normal expression of GT198 is mostly nuclear and decreases during development

**Figure 2:**
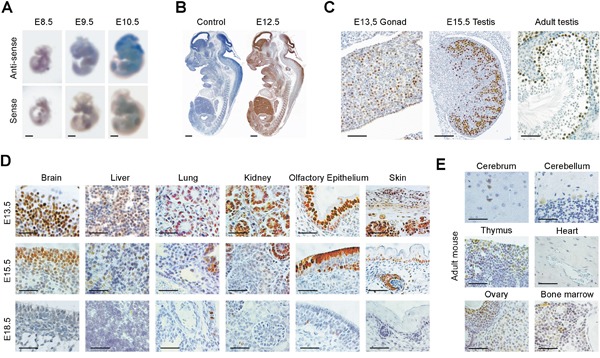
Normal GT198 expression in mouse tissues **(A)** Whole mount *in situ* hybridization showing GT198 mRNA stained in blue at mouse embryonic stages of E8.5, E9.5 and E10.5. **(B)** Immunohistochemical staining of GT198 protein in mouse embryo at stage E12.5. A negative control absence of primary antibody is at the left. **(C)** GT198 protein expression in developing and adult mouse testes showing nuclear staining in germ cells. **(D)** Decreased GT198 expression in multiple tissues as indicated in mouse developmental stages E13.5, E15.5, and E18.5. **(E)** Positive expression in adult mouse brain, thymus, ovary, bone marrow but not in heart. Immunohistochemical staining sections **(B-E)** are counter-stained with hematoxylin. Scale bars = 1 mm **(A-B)**, 50 μm **(C-E)**.

GT198 expression in normal tissues is essential to assess its abnormal patterns in cancer, and is indispensible to predict therapeutic side effects, should GT198 be a drug target. Here, we systematically characterized GT198 expression using normal mouse tissues. GT198 was previously shown to increase in primitive ectoderm during embryonic stem cell differentiation [39]. During mouse embryonic development, GT198 mRNA revealed by *in situ* hybridization showed a marked increase in brain at embryonic stages from E8.5 to E10.5 (Figure 2A), with a peak in protein expression at E12.5 in all three germ layers (Figure 2B). This pattern is consistent with the previous Northern blot analysis [42]. GT198 expression was rapidly downregulated in most tissues and major organs from E13.5 to E18.5, except in testis where spermatocytes were continuously positive (Figure 2C-2D). In adult mice, other than testis, low level of GT198 expression was also detected in the ovary, thymus, bone marrow, and certain types of neurons in brain (Figure 2E). Blood vessels in the heart and lung were negative in expression (Figure 2D-2E). Except for normal ovarian corpus luteum and certain neural cells, GT198 protein expression was mostly nuclear in normal tissues. This was in sharp contrast to the cytoplasmic expression observed in human cancer (Figure 1A). The overall expression pattern of GT198 resembles a class of proteins called *cancer-testis antigens with the expression in cancer, embryo, testis, but not in most adult tissues*. GT198 expression is also remarkably similar to that of BRCA1 and BRCA2 expression in mice [47]. *The developmental decrease of GT198 reflects its functional involvement in stem or progenitor cell differentiation. Its re-expression in cancer infers a dedifferentiation status*.

### GT198^+^ pericytes give rise to tumor cells in human oral cancer

**Figure 3:**
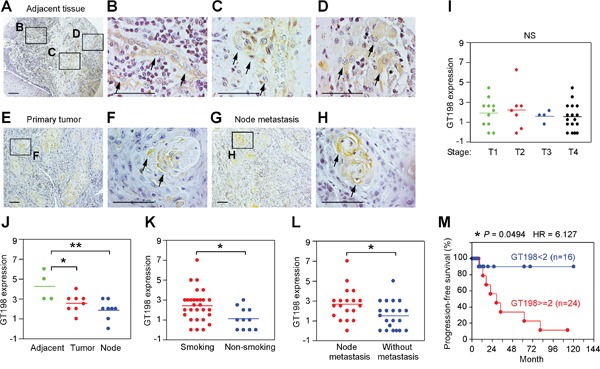
GT198^+^ pericytes give rise to tumor cells in human oral cancer Immunohistochemical staining of GT198 in 40 cases of human oral cancer. **(A-D)** In tumor adjacent tissues, GT198^+^ pericytes are thickened **(B)**, detached from vessels **(C)**, and overgrown into GT198^+^ tumor nodules **(D)**. **(E-F)** GT198 positive nodules in primary cancer. **(G-H)** Positive nodules in lymph nodes. Boxed areas are enlarged. Arrows indicate GT198^+^ cells. **(I)** GT198 expression is found in all stages of tumor. **(J)** Increased GT198 expression is associated with tumor adjacent tissues; **(K)** with tumors from smokers versus non-smokers; and **(L)** with tumors from patients with lymph node metastasis. **(M)** Increased GT198 expression is associated with decreased progression-free survival of oral cancer patients. n, number of cases in analyzed groups. *P* values are calculated by t test in **(I-L)** and by log-rank test in **(M)**.

**Figure 4:**
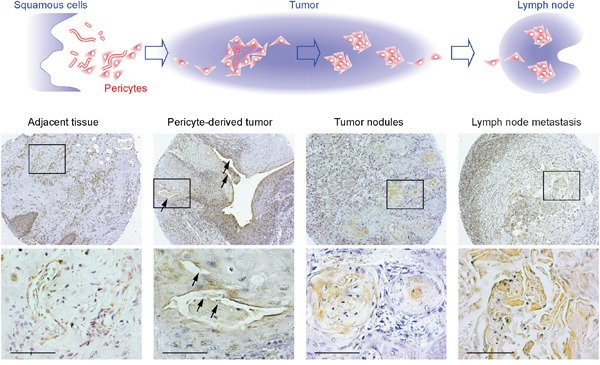
GT198^+^ pericytes give rise to pericyte-derived tumor cells which migrate into lymph node Immunohistochemistry staining of GT198 showing the progression steps of GT198^+^ pericytes from tumor adjacent tissues, to pericyte-derived functional tumor vessels, to tumor nodules absent of vessel cavities, and to tumor nodules in lymph nodes. Hypothetic models are shown at the top of the corresponding staining images. Boxed areas are enlarged below. Arrows indicate the locations where red blood cells are present in tumor-enclosed vessels. Sections are counter-stained with hematoxylin. Scale bars = 100 μm.

Using GT198 cytoplasmic expression as a marker, we analyzed 40 cases of human oral cancers with associated clinical information (Supplementary Table 1). In angiogenic tissues adjacent to tumor, pericytes in capillaries were thickened, and overexpressed cytoplasmic GT198 (Figure 3A-3B). These GT198^+^ pericytes further proliferated into small tumor nodules and detached from blood vessels (Figure 3C-3D). In advanced tumor, the GT198^+^ pericytes in nodules continued to differentiate into tumor cells (Figure 3E-3F). Pericyte-enclosed nodules at the early stage were still functional vessels with red blood cells in their cavities (Figure 4), which is consistent with the observations from others that tumor produces its own vessels [27]. In lymph node metastasis, pericyte-derived GT198^+^ nodules were found in the lymph nodes (Figures 3G-3H and 4). These observations suggest that pericytes overexpressing GT198 have malignant potential and give rise to tumor cells which are capable to migrate into lymph nodes.

GT198 expression in pericytes or in their derived tumor cells was found in all stages of oral tumors analyzed in 40 patients (Figure 3I). However, the expression scores were higher in tumor adjacent tissues where angiogenic blood vessels were abundant (Figure 3J). GT198 expression levels in tumor were higher in smokers versus non-smokers (Figure 3K), and were positively associated with lymph node metastasis (Figure 3L). When patients were divided into two groups by GT198 expression scores, higher level of GT198 expression was significantly associated with decreased progression-free survival in patients (HR = 6.127, 95% CI) (Figure 3M), suggesting that the presence of GT198^+^ pericytes promotes tumor progression and is a worse prognostic indicator in oral cancer patients.

**Figure 5:**
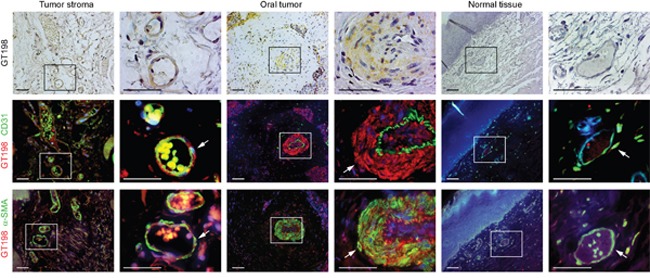
GT198^+^ pericytes in CD31^+^ vessel-derived oral tumor Three serial cut sections of oral tumor and adjacent normal tissues were analyzed for pericytes. Immunohistochemical staining of GT198 is shown in the top panels counter-stained with hematoxylin. Fluorescent double staining of GT198 in red, CD31 and α-SMA in green, and DAPI in blue, is shown in the middle and bottom panels, respectively. Each enlarged boxed area is shown at the right. In tumor stroma, GT198^+^ α-SMA^+^ pericytes enclose a thin layer of CD31^+^endothelial cells. In tumor, CD31^+^endothelial layer is enclosed by GT198^+^ pericytes, which further proliferate into tumor cells (Supplementary Figure 2). In normal vessels, GT198 expression is largely negative. Arrows indicate pericytes. Scale bars = 100 μm.

To further confirm that GT198^+^ vessels give rise to tumor cells in human oral cancer, we co-stained GT198 together with CD31 for endothelial cells or α-SMA for pericytes or perivascular cells using adjacent tissue sections (Figure 5). In angiogenic tumor stroma, GT198 was co-stained with α-SMA^+^ pericytes enclosing a thin layer of CD31^+^ endothelium (Figure 5 left panels). Within the tumor, CD31^+^ endothelium was still intact and was surrounded by a thickened layer of GT198^+^α-SMA^+^ perivacular cells (Figure 5 middle panels). At this stage, its vessel cavity had already infiltrated with tumor cells. When tumor progressed, the vessel structure disintegrated with a gradual loss of CD31^+^ endothelial layer and proliferation of perivascular cell-derived GT198^+^ tumor cells (Supplementary Figure 2). In contrast, GT198 expression in pericytes was mostly negative in normal oral tissues in the absence of angiogenesis (Figure 5 right panels). These results indicate that GT198^+^ pericytes in angiogenic blood vessels are capable to give rise tumor cells in human oral cancer.

### GT198^+^ pericytes give rise to tumor cells in U-251 glioblastoma xenografts

**Figure 6:**
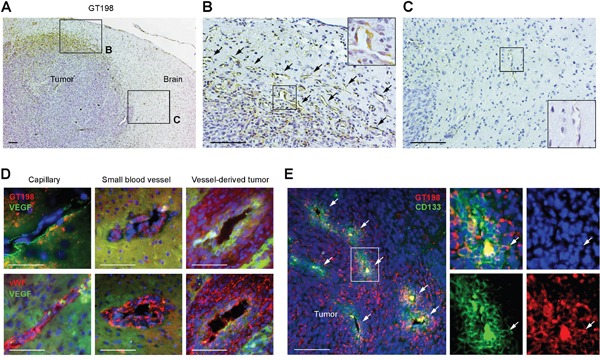
GT198^+^ pericytes give rise to tumor cells in U-251 glioblastoma xenografts **(A)** Immunohistochemical staining of GT198 showing pericyte-specific staining at the periphery of the tumor. Angiogenic area is enlarged in **(B)** and normal rat brain area in **(C)**. **(B)** GT198^+^ pericytes in angiogenic vasculature. Arrows indicate GT198^+^ pericytes. **(C)** Normal capillary absence of GT198 expression in rat brain adjacent to tumor. Boxed area is enlarged. **(D)** Immunofluorescent double staining of GT198 or vWF in red, and VEGF in green, in pericyte-enclosed vessels of different sizes. **(E)** Immunofluorescent double staining of GT198 in red, and CD133 in green, showing enriched stem cell populations near vessel lumens of the merging vessels. White arrows indicate vessel lumens and a double-positive cell in the enlarged area. Scale bars = 100 μm.

To investigate malignant pericytes in brain tumor, we examined U-251 human gliobastoma intracranial xenografts in rat brain which are characterized by extensive microvascular proliferation in tumor angiogenesis. We found high level of GT198 expression associated with angiogenic pericytes in tumor but not with quiescent pericytes in normal vessels (Figure 6A-6C). In particular, GT198 expression is cytoplasmic consistent with an active GT198 status (Figure 6B) [39]. Compared to the surrounding GT198^-^ normal vessels in rat brain (Figure 6C), GT198^+^ pericytes were specific to angiogenic vessels at the periphery of the developing tumor (Figure 6A). The GT198^+^ vessels also expressed VEGF, vWF (Figure 6D), as well as pericyte marker α-SMA (Supplementary Figure 3). Importantly, the stem cell marker CD133 was co-expressed with GT198 in cells surrounding the vessels (Figure 6E). It appeared that GT198^+^ pericytes are able to proliferate as well as differentiate to produce larger vessels out of smaller capillaries (Figure 6D). The larger vessels further merge into a tumor mass leaving original vessel lumens in the middle of the tumor (Figure 6E). Tumor cells surrounding vessel lumens were undifferentiated expressing CD133, while cells distal to the lumens were more differentiated lacking CD133 (Figure 6E). These observations suggested that GT198^+^ pericytes are derived from tumor stem cells or progenitors and are able to proliferate and differentiate back into tumor cells, utilizing vessel formation during the process, so that continued cycles expand tumor size. This conclusion is in part consistent with the observations from others on vascular mimicry of tumor-derived vessels. Our data indicate that GT198^+^ pericytes are both angiogenic and tumorigenic.

### Vascular co-option between human GT198^+^ pericytes and rat endothelial cells

**Figure 7:**
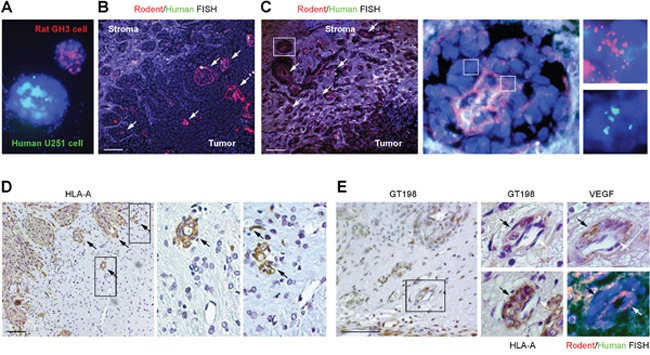
Vascular co-option between human pericytes and rat endothelial cells **(A)** Validation of human chromosome painting FISH probe in green, rodent probe in red, and DAPI in blue using human U-251 and rat GH3 cells. **(B-C)** FISH analysis of rat endothelial cell layers in red (arrows) enclosed by human tumor cells. Non-specific signals are in white due to the overlay of red and green. Boxed area of an enlarged vessel shows disintegrated rat endothelial layer in red enclosed by human cells in green. **(D)** Immunohistochemical staining of human specific HLA-A, with boxed areas enlarged showing rat capillaries captured by human pericytes. Arrows indicate pericytes. **(E)** Immunohistochemistry and FISH analyses using adjacent sections. GT198^+^ pericytes (black arrows) are HLA-A^+^ and VEGF^+^, and enclose rat endothelial cells (white arrows). Scale bars = 100 μm.

The above observation raised a question about the relationship between GT198^+^ pericytes and endothelial cells in this xenograft tumor model. To examine the origins of both cell types, we performed FISH analysis using dual-colored human and rodent FISH probes to distinguish cell origins. Human and rodent chromosome painting probes were first validated and found specific to human or rodent cells (Figure 7A and Supplementary Figure 4A). Subsequent FISH analysis showed that the rat cell-derived endothelial layer in red was surrounded by human tumor-derived cells, with smaller vessels at the tumor periphery and large fused vessels at the center of the tumor (Figure 7B-7C). In larger vessels, the endothelial layer was disorganized (Figure 7C and Supplementary Figure 5). Although the human FISH probe in green was weak, it was clear that these human cells lacked red signal. Red signal was also found in normal rat brain tissue adjacent to tumor (Supplementary Figure 4B). The human origin of pericytes or tumor cells was also confirmed by immunohistochemical staining using a human-specific antibody against the human leukocyte antigen HLA-A, in which human HLA-A^+^ pericytes captured rat HLA-A^-^ endothelial cells (Figure 7D). Using adjacent sections, we also confirmed that GT198^+^ pericytes were both VEGF^+^ and HLA-A^+^, enclosing a thin layer of rat endothelium stained in red by rat FISH probe (Figure 7E). Human tumor cells stained in green were able to infiltrate into rat tissues stained in red (Supplementary Figure 4C). Together, these data suggest that the angiogenic vessels in U-251 xenograft tumors are mosaic, containing human tumor-derived pericytes and rat host-derived endothelial cells. Therefore, vessel co-option and vascular mimicry may result from a single process where tumor-derived GT198^+^ pericytes adopt the host's endothelial layer to differentiate into tumor cells via the process of angiogenesis.

### GT198 activation stimulates VEGF and promotes tube formation in U-251 cells

**Figure 8:**
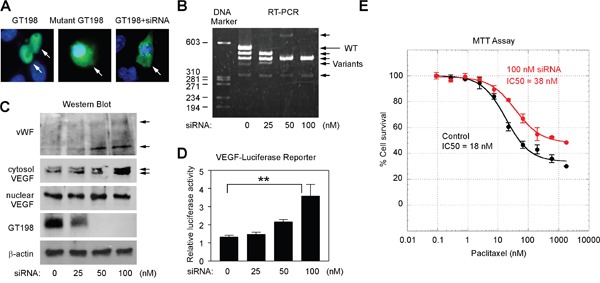
GT198 activation stimulates VEGF and increases U-251 cell survival **(A)** U-251 cells were transfected with GFP-GT198 (1-217), GFP-mutant GT198 (126-217), or together with GT198 siRNA (100 nM) as indicated. **(B)** GT198 siRNA switched the balance of expression between the wild type (long arrow) and GT198 splice variants (short arrows) in RT-PCR analysis. **(C)** GT198 siRNA induced cytosolic VEGF isoforms and vWF expression (arrows) in Western blot analysis. Reduced wild type GT198, unchanged nuclear VEGF, and β-actin served as controls. **(D)** U-251 cells were cotransfected with VEGF promoter-luciferase (100 ng) together with GT198 siRNA. Relative luciferase light units were shown as means of triplicate transfections ± S.E. (*n*=3). *P* values shown were calculated by linear progression. **(E)** U-251 cells were transfected in duplicate with 0 nM (black) or 100 nM GT198 siRNA (red), and treated at indicated concentrations of cytotoxic drug paclitaxel for 72 hours before analysis using MTT cell viability assay. IC50 values are calculated by nonlinear regression sigmoidal curve fit.

The U-251 cell-derived GT198^+^ pericytes are clearly not quiescent. To test if cytoplasmic GT198 expression activates the pericytes, we utilized a previously characterized siRNA that suppresses wild type GT198 but stimulates its active splice variants [39]. We have previously shown that GT198 alternative splice variants induce cytoplasmic translocation of the wild type [40], and stimulate target genes including VEGF [41]. In U-251 cells, while GFP-wild type GT198 (1-217 aa) was nuclear and GFP-mutant GT198 (126-217 aa) was cytoplasmic, the siRNA induced cytoplasmic translocation of the wild type (Figure 8A). We then tested this siRNA by transfection of U-251 cells to evaluate marker gene expression during GT198 activation. We found that the siRNA induced a switch in alternative splicing balance by diminishing wild type GT198 and increasing its active splice variants (Figure 8B), and also induced cytosolic VEGF and vWF isoforms expression (Figure 8C). As controls, nuclear VEGF expression detected by the same anti-VEGF antibody was unchanged which may represent a less angiogenic VEGF isoforms in nucleus [48]. Wild type GT198 was downregulated by its siRNA as expected (Figure 8C), the expression of cytosolic GT198 was too low to be detected by Western blot although can be detected by fluorescent imaging (Figure 8A). In addition, using a human VEGF-luciferase reporter [41], we found that GT198 siRNA stimulated the VEGF promoter in U-251 cells (Figure 8D). Furthermore, GT198 siRNA increased U-251 cell survival when tested under increasing concentrations of a cytotoxic drug paclitaxel in an MTT assay (Figure 8E). In siRNA-transfected U-251 cells, both efficacy and sensitivity of paclitaxel cytotoxicity were reduced (Figure 8E). Together, these results indicate that activation of GT198 induced angiogenic factors including VEGF and promoted cell survival in U-251 cells.

**Figure 9:**
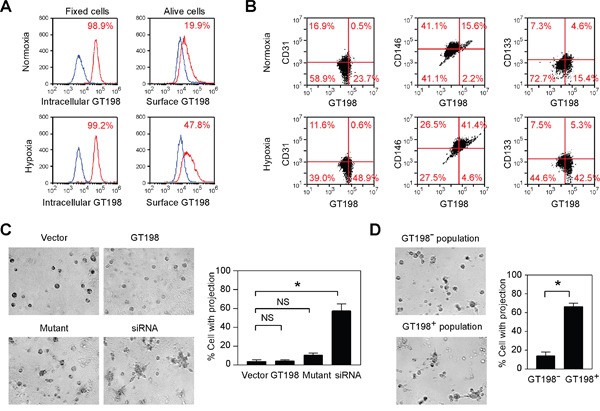
Hypoxia induces cell surface expression of GT198 and GT198 activation promotes tube formation in U-251 cells **(A)** Hypoxia-induced U-251 cells were FACS analyzed using either methanol fix for intracellular GT198, or living cells for surface GT198. **(B)** Flow cytometry quadrant dot plots with GT198 on the x-axis and CD31, CD146, and CD133 as indicated on the y-axis. The numbers at each corner indicate the percentage of U251 cells. **(C)** U-251 cells were transfected with wild type GT198 (1-217), mutant GT198 (126-217), siRNA (100 nM), or vector as a control. Tube formation on Matrigel cultured in serum-free medium overnight was photographed, and the percentage of cells with projections was quantified (n=2). **(D)** GT198^+^ and GT198^-^ cell populations isolated from siRNA-transfected U-251 cells were compared in Matrigel tube formation assays with quantification. *P* values were calculated by unpaired two-tailed t test. * *P*<0.05.

Hypoxia is a well-characterized condition to induce angiogenesis. Using FACS analysis, we surprisingly found that GT198 protein was detectable on living U-251 cell surface, and was markedly increased in hypoxia (Figure 9A). Thus, GT198 is capable to translocate into cytoplasm as well as to cell surface, given that translocation among cellular compartments is a well-observed phenomenon in many oncoproteins such as p53 [49], Rb [50], BRCA1 [51], and EWS [52]. Taken the advantage of surface GT198 in FACS analysis, we found that hypoxia induced an increase of pericyte marker CD146, in contrast to CD31 or CD133, in GT198^+^ cell population (Figure 9B). This result supports the expression of GT198 in pericytes in U-251 cells. In addition, when U251 cells were transfected with wild type GT198, mutant GT198, or siRNA, only siRNA induced detectable tube formation on Matrigel in serum-free medium (Figure 9C). Mutant-transfected cells were apoptotic [39], and thus did not induce tube formation. The activity in siRNA-transfected cells was potentially through the stimulation of VEGF (Figure 8C). The isolated GT198^+^ population from siRNA-transfected cells also showed increased tube formation than GT198^-^ population (Figure 9D). Taken together, our results support that activation of GT198 promotes vasculogenic activity in U-251 cells.

### GT198 vaccination suppresses GL261 glioma growth in mouse tumor model

**Figure 10:**
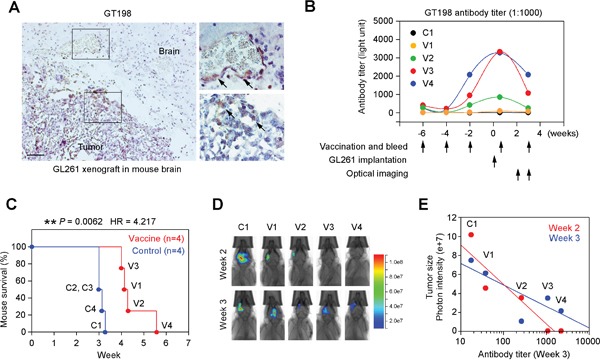
GT198 vaccination suppresses mouse GL261 glioma growth **(A)** Immunohistochemical staining of GT198 in C57BL/6 mouse brain with GL261 glioma xenograft showing positive angiogenic vessels and tumor cells. Arrows indicate GT198^+^ cells. **(B)** Mice were vaccined by subcutaneous injection of recombinant GT198 protein as antigen (100 μg/injection) at indicated time points starting 6 weeks before GL261 tumor implantation. Tail blood serum (1:1000) from each bleed was analyzed for anti-GT198 antibody titer. One control and 4 vaccined mice are shown. **(C)** Kaplan-Meier curve analysis of mouse survival in control and vaccine groups (n=4) implanted with GL261. *P* and HR values are calculated by log-rank test. **(D)** Optical bioluminescent imaging of living mice at week 2 and week 3. **(E)** Tumor size as a function of GT198 antibody titer (log scale). Decreased tumor sizes, represented by photon intensity, are correlated with increased antibody titer particularly at the early stage in week 2.

The cell surface expression of GT198 may permit targeted immunotherapy against angiogenesis, and we tested this potential in a mouse brain tumor model implanted with syngeneic GL261 glioma. GT198 was strongly expressed in a proportion of tumor cells and vessel pericytes of GL261 tumor implanted in C57BL/6 mouse brain (Figure 10A). Mice were subcutaneously vaccined with repeat doses of denatured recombinant GT198 protein as antigen, or PBS as control, starting at six weeks before tumor implantation (Figure 10B). Anti-GT198 antibody titer measured from each bleed showed that the antibody peaked around the time of tumor implantation but with marked individual variability in the vaccined mice, while the PBS control mice lacked anti-GT198 antibody (Figure 10B). The control group (n=4) died of tumor between 21-23 days after tumor implantation, whereas the vaccine group (n=4) died between 28-39 days, with significantly prolonged survival time (Figure 10C). The V4 vaccined mouse had highest antibody titer and survived longest. Optical imaging of tumor sizes suggested a reciprocal correlation between anti-GT198 antibody titers and the tumor sizes (Figure 10D-10E), particularly at the early stage of tumor development at week 2. Our results suggested that anti-GT198 antibody, if successfully produced by vaccination, may have protective effect against GL261 tumor growth and prolong mouse survival.

We also evaluated GT198 vaccination using primed dendritic cells in a mouse tumor model implanted with 4T1 breast tumor containing GT198^+^ pericytes and GT198^+^ stromal cells (Supplementary Figure 6A). GT198 protein-primed matured dendritic cells inhibited tumor growth without metastasis, while the control mice showed metastasis (Supplementary Figure 6B-6D). Although more *in vivo* stimuli may be present during angiogenesis, the above data together support that cytoplasmic GT198 is a marker for activated pericytes which have malignant potential to recapitulate tumor.

## DISCUSSION

Solid tumor growth is often dependent on angiogenesis. Despite intensive studies on the cellular and molecular basis of angiogenesis [4], transient clinical benefit and drug resistance to anti-angiogenic therapies in patients indicate the need of new knowledge [6, 53, 54]. Using the oncoprotein GT198 as a marker, we report here that malignant pericytes expressing GT198 give rise to tumor cells through the process of angiogenesis. The finding of GT198^+^ pericytes provides a strengthen link among existing angiogenesis models. In a possible scenario, sprouting angiogenesis may be initiated by surrounding malignant pericytes, which produce higher than normal levels of VEGF (Figure 7E and 8C) to stimulate the sprouting of endothelial tip cells growing toward the pericytes. Next, malignant pericytes capture, adopt, and enclose incoming endothelial cells as vessel co-option (Figure 7B-7D). Then, intussusception is promoted by the abundance of non-quiescent malignant pericytes (Figure 6B), due to the lack of quiescent normal pericytes to maintain vessel stability. Thereafter, proliferation and differentiation of tumor cells derived from pericytes result in thick-walled large vessels (Figure 6D), which have previously been described as vascular mimicry. Furthermore, CD133^+^ cancer stem cells can differentiate into malignant pericytes in tumor vessels [25], since tumor cells and pericytes are interchangeable (Figure 6E). Finally, it is possible that bone marrow-derived myeloid stem/progenitor cells may differentiate into pericyte progenitors. Evidence from others also supports the incorporation of bone marrow cells into perivascular locations rather than into vessel lumens [21, 55]. Our study does not exclude the possibility of the existence of tumor-derived endothelial cells [20, 24], or non-endothelial lined vessels [18, 19], but emphasizes that tumor-derived pericytes are more evident, at least in the models studied here. Specifically, we provide strong evidence to show that the endothelial cell layer is derived from the host in U-251 xenografts (Figure 7B-7C and Supplementary Figure 5). The host-derived endothelial layer is gradually lost into disintegration and is ultimately overgrown by tumor cells (Figure 7B-7C). Together, these studies suggest that previous hypotheses in the field are not mutually exclusive; rather, they are multiple steps in a linked process mediated by malignant pericytes during the process of angiogenesis.

**Figure 11:**
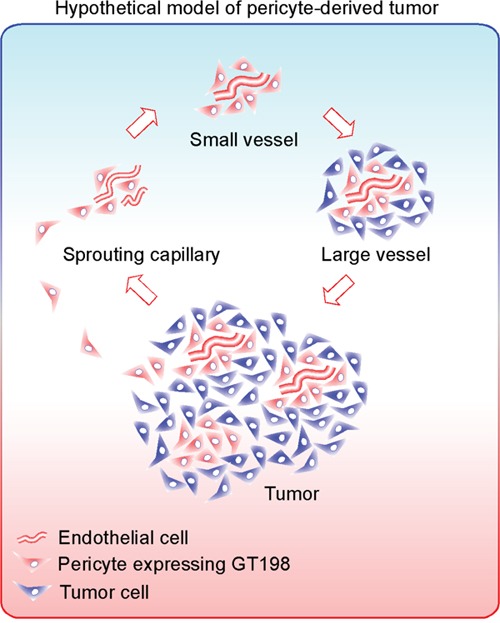
Hypothetical model of pericyte-derived tumor An integrated hypothetic model showing that malignant pericytes expressing GT198 (red) capture sprouting endothelial cells (red tubing) to form small blood vessels. Pericytes are progenitors which are capable to proliferate and differentiate into tumor cells (blue) in larger vessels. The large vessels further merge into tumor containing GT198^+^ nodules. Through angiogenic vessel formation, the continued pericyte-tumor cell cycles expand the tumor. The net effect of this process is differentiation from malignant pericytes into tumor cells facilitated by angiogenesis.

An integrated hypothetical model is presented in Figure 11. First, malignant GT198^+^ pericyte progenitors capture sprouting capillaries to form smaller vessels. Continued differentiation from pericytes into tumor cells yields larger tumor-enclosed vessels that are still functional. The fusion of these vessels produces tumor masses containing GT198^+^ tumor nodules, in which pericyte progenitors continue the cycle. In support of this hypothetic model, tumor-derived pericytes have been previously demonstrated [25, 26]. Tumor cells with migratory pericyte behavior have been described in details [56]. Pericyte-derived tumor cells were also observed in reports of vascular mimicry or tumor-do-it-yourself vessels [27], or even as the product of reversible epithelial to mesenchymal transition [57]. Here we extend these previous observations using GT198 as a marker to confirm that these two processes are actually interconnected in concurrent cycles during tumor angiogenesis.

When tumor cells are already present, such as in metastatic tumors or in xenografts, malignant pericytes can be tumor-derived. In contrast, in early human primary cancers before tumor growth has occurred, malignant GT198^+^ pericytes could be derived from normal pericytes, such as through acquisition of somatic mutations [41]. Thus, distinct initiating stimuli would lead to the development of malignant pericytes as a common step in tumor angiogenesis. Our observations on migrating GT198^+^ pericytes in human oral cancer (Figure 4), are consistent with this hypothesis. These findings also provide direct evidence to support the notion that during the process of epithelial-to-mesenchymal transition, mesenchymal stromal cells become malignant pericytes [26], and are thereby able to migrate.

The existence of malignant pericytes may have implications for the effectiveness of current anti-cancer strategies. For example, the development of radiation resistance in tumors, may be explained by the survival of GT198^+^ malignant pericytes; since activation of GT198 stimulates DNA repair [37, 39], which could support the survival of resistant cells. Similarly, the transient effect of anti-VEGF therapy is often due to the development of drug resistance. The VEGF target may not be specific enough to completely suppress the tumor vasculature [53]. While VEGF^+^ pericytes may be inhibited, VEGF^-^GT198^+^ pericytes would be predicted to escape anti-VEGF therapy, particularly in advanced or metastatic tumors [58]. Based on our preliminary evidence (Figure 10), anti-GT198 in combination with anti-VEGF therapy may be one practical approach for overcoming such resistance.

In summary, our study shows that overexpression of cytoplasmic GT198 in malignant pericytes is a common feature of the angiogenic tumor vasculature among various human primary cancers and rodent tumor models. By analyzing human oral cancers and glioblastoma xenografts, we suggest that malignant pericytes expressing GT198 give rise to tumor cells. These observations imply that existing hypotheses in tumor angiogenesis are interconnected and reconcilable when malignant pericytes are incorporated. GT198 protein has the potential to be a new target in anti-angiogenic therapy.

## MATERIALS AND METHODS

### Human cancer sections

Institutional Review Board approval was obtained following institutional guidelines using de-identified human cancer formalin-fixed paraffin-embedded (FFPE) sections from Augusta University, Augusta, GA. Human oral tumor microarray FFPE sections containing 40 cases of triplicate cores at 1.0 mm and associated clinical information were originally prepared by the Head and Neck Cancer SPORE at University of Pittsburgh Cancer Institute, Pittsburgh, PA. Additional common human tumor FFPE microarrays at 2.0 mm in diameter were from Imgenex Corporation, San Diego, CA. Pathology diagnosis was verified through histological examination before immunohistochemistry analysis.

### Xenograft mouse tumors

FFPE sections of xenograft tumors were originally prepared using human cancer cell lines xenografted into nude mice. The six human cancer cell lines include breast cancer BT474, ovarian cancer OVCAR-3, glioblastoma T98G, lung cancer Calu-6, prostate cancer DU145, and colon cancer SW480.

### Whole mount *in situ* hybridization

Mouse embryos (FVB/N) at stages E8.5, E9.5, and E10.5 were fixed overnight in 4% paraformaldehyde in PBS with 0.1% Tween at 4°C and dehydrated through serial methanol at 25%, 50%, 75% and 100%. The dehydrated embryos were treated with RNase-free DNase I (50 U/ml) before hybridization with denatured riboprobe. The antisense and sense riboprobes were produced by *in vitro* transcription in the presence of Digoxigenin-UTP (Roche Diagnostics) using full-length GT198 cDNA in the pcDNA3 vector. Stained embryos were fixed and photographed.

### U-251 glioblastoma xenograft in rat brain

The rat xenograft tumor model of U-251 glioblastoma was previously described [59]. The human U-251 cell line was authenticated with the following short tandem repeat and Amelogenin profiles matched: HT01, D5S818, D13S317, D7S820, D16S539, CSF1PO, vWA, and TPOX. Animal protocols were approved by the IACUC at Augusta University. NIH-RNU nude rats (Charles River Laboratories, Frederick, MD) at five weeks of age were anesthetized and injected with 5 μl of U-251 cells (4×10^5^). Briefly, the surgical zone was swabbed before incision, and a 3 mm hole in the skull was made by a dental drill. U-251 cells were stepwise injected at a rate of 0.5 μL per 30 second. The surgical hole was sealed with bone wax after the procedure. Rats were sacrificed on day 22. Tumors were harvested to prepare FFPE sections for immunohistochemistry, immunofluorescence, and fluorescent *in situ* hybridization (FISH) analyses.

### Immunohistochemistry

Polyclonal rabbit antibody against GT198 was affinity purified and previously described [41, 42]. FFPE sections or tumor microarrays were deparaffinized and dehydrated through xylene and ethanol series, followed by antigen retrieval in 10 mM sodium citrate buffer, pH 6.0, containing 0.05% Triton at 90°C for 20 min. Anti-GT198 (1:200), anti-VEGF (RB-9031, Thermo Scientific, Fremont, CA), and anti-HLA-A (ab70328, Abcam, Cambridge, MA) were incubated at 4°C overnight. Antibody binding was detected using biotinylated secondary antibody followed by detecting reagents (Abcam, Cambridge, MA). Sections were counterstained with hematoxylin. Immunohistochemistry staining of human oral cancers was scored in each 1.0 mm core as 0, completely negative; 0.5, <10 positive cells; 1, 11-100 positive cells; 2, 101-1000 positive cells; 3, >1000 positive cells. The scores obtained from 3 triplicate cores in each patient were added as final scores, ranging 0-7 (Supplementary Table 1). Scored data was graphed by scattergram or Kaplan-Meier curves using GraphPad Prism software.

### Fluorescent *in situ* hybridization (FISH)

FISH analysis was carried out using FFPE sections of U-251 glioblastoma xenograft tumors in rat brain. Immunohistochemical staining of GT198, VEGF and HLA-A using adjacent sections was carried out for comparison. Paraffin-embedded rat brain tissue sections were deparaffinized through xylene and ethanol series, followed by antigen retrieval as described above. The sections were further digested by 5% pepsin in 0.01 N HCl at 37°C for 10 min before use. Gene-specific or locus-specific FISH probe signals were found weak due to higher background signals in FFPE sections, so that only chromosome painting FISH probes were used in this study. The rodent-specific chromosome painting probe was a Cy3-labeled mouse painting probe in red (1200-11MCy3-02, StarFISH, Cambridge, UK), which detected both mouse and rat but not human nuclei. The human-specific chromosome painting probe was prepared by labeling purified human placenta genomic DNA in green using SpectrumGreen dUTP (Nick Translation Kit, Vysis) following the manufacturer's protocol. Prior to FISH analysis, probe specificity was validated using mixed human U-251 and rat GH3 cells, or human HeLa and mouse P19 cells. Subsequently, rodent and human probes were applied simultaneously on rat brain tumor sections for dual-color visualization. Sections were hybridized using Vysis reagents and counterstained with DAPI II before visualization using fluorescence microscopy.

### Immunofluorescence and GFP transfection

Paraffin-embedded rat brain tissue sections were deparaffinized through xylene and ethanol series, followed by antigen retrieval as above. Immunofluorescence double staining was carried out in 1% horse serum using rabbit anti-GT198, or rabbit anti-von Willebrand factor (vWF) (A0082, DAKO, Carpinteria, CA), together with mouse anti-VEGF (sc-7269, Santa Cruz Biotechnology, Dallas, TX), or mouse CD31 (550274, BD Biosciences, San Jose, CA) or mouse α-SMA (A2547, Sigma, St. Louis, MO). CD133 was detected using rabbit anti-CD133 (ab19898, Abcam, Cambridge, MA) together with mouse anti-GT198 (1:200, Novus Biologicals, Littleton, CO). Secondary antibodies were anti-mouse or anti-rabbit Alexa Fluor-conjugated antibodies (Invitrogen, Carlsbad, CA). Sections were counterstained with DAPI. In GFP transfection assays, GFP fusions of wild type GT198 (1-217) or mutant (126-217) were transfected into U-251 cells in chamber slides. GT198 activation was induced by cotransfection of 100 nM of GT198 siRNA 5’-GUGAGUGAUGCUGACCUUCA-3’. The siRNA induces GT198 splice variants by targeting exon 4 as previously described [39, 40]. GFP-GT198 in the cytoplasm was detected by fluorescence and countered stained with DAPI.

### Western blot and reverse-transcript PCR

U-251 cells were maintained in DMEM supplemented with 10% fetal bovine serum, 2 mM glutamine, 100 U/ml penicillin and 0.1 μg/μl streptomycin, and incubated in 5% CO2 at 37°C. Cells were transfected in 6-well plates by Lipofectin (Invitrogen, Carlsbad, CA) using 0-100 nM GT198 siRNA as indicated. After 16 hours, the cytosolic fraction was isolated using lysis buffer (20 mM HEPES, pH 7.4, 10 mM KCl, 1 mM EDTA, 1 mM EGTA, 1 mM DTT, 10 μg/ml protease inhibitors) with the addition of 1% Triton X-100 for 15 min on ice, and was probed by rabbit anti-VEGF (RB-9031) and anti-vWF. For the nuclear extract fraction, cells were lysed in the above lysis buffer with 0.05% Triton X-100 for 15 min on ice. The nuclei were then collected by centrifugation and further lysed in the above buffer with 420 mM NaCl for 30 min. Nuclear extracts were probed by anti-VEGF, anti-GT198, and anti-β-actin (Sigma, St Louis, MO). Western blots were detected using the ECL system (GE Healthcare, Piscataway, NJ). For reverse-transcription PCR, total RNA from siRNA-transfected U-251 cells was isolated by Trizol and analyzed using one-step RT-PCR kit (Qiagen, Valencia, CA). GT198 primers used are at Exon 1 and 5: 5’-CTTCCCCTTCAGCCAATCAC-3’ and 5’-GGTAGCTGCTTTAATGTTCTTCAA-3’.

### VEGF promoter luciferase assay and MTT assay

Human VEGF promoter luciferase reporter (-1970 to +19) was previously described [41]. U-251 cells were transfected in triplicate in 24-well plates using 0.1 μg/well VEGF-luciferase reporter together with GT198 siRNA (0-100 nM), as indicated. Cells were harvested 16 hours after transfection, and relative luciferase activities were measured by a Dynex luminometer. Data are presented as means of triplicate transfections ± standard errors. For MTT assay, U-251 cells were transfected in duplicate in 48-well plates using 0 nM or 100 nM GT198 siRNA, and incubated for 72 hrs using increasing concentrations of paclitaxel (0, 0.27, 0.82, 2.4, 7.4, 22, 66, 200, 600, 1800 nM). The MTS reagent (G3580, Promega) was diluted 1:10 in phenol red-free medium and incubated at 200 μl/well for 30 min at 37°C. The reacted colored medium was transferred to flat-bottom clear 96-well plates and the absorbance at 490 nm was determined using a Tecan Safire microplate reader.

### FACS analysis and tube formation assay

FACS analysis and data acquisition were performed on a flow cytometer (Accuri C6, BD Biosciences). Hypoxia U-251 cells were generated in DMEM with 10% fetal bovine serum, in 1% oxygen and 5% CO2 at 37°C overnight. Living normoxia and hypoxia U-251 cells were stained in PBS with 1% BSA on ice using anti-GT198 and anti-rabbit Alexa 448; or co-stained with PE-conjugated anti-CD31 or CD146 (342004, BioLegend, San Diego, CA), or CD133 (Miltenyi Biotech, Germany). For fixed cells, cells were incubated in 1% paraformaldehyde for 10 min and methanol for 20 min on ice before antibody staining. A minimum of 10,000 cells within the gated region were analyzed.

In tube formation assay, U-251 cells were lipofectin-transfected for 4 hours in 6-well plates using wild type GT198 (1-217), mutant GT198 (126-217), GT198 siRNA (100 nM), or pcDNA3 vector as a control. After overnight culture, transfected cells were seeded at 2×10^4^ per well in duplicate in a 96-well plate containing 50 μl Matrigel in serum-free medium, and incubated for overnight before photograph and quantification. To isolate GT198^+^ cell population, siRNA-transfected U-251 cells (2×10^5^) were incubated with anti-GT198 antibody (1:100, 1 ml) and protein A agarose beads (30 μl). Bound cells were eluted by His-tagged GT198 protein (3 μg/ml, 200 μl). Unbound cells (GT198^-^) and eluted cells (GT198^+^, 28% by cell count) were washed and subjected to the tube formation assay.

### GT198 vaccination in GL261 and 4T1 mouse tumor models

For GL261 glioma model, denatured GT198 antigen for vaccination was produced from insoluble inclusion body of recombinant GST-GT198 protein isolated from E. coli BL21(DE3), since insoluble antigen has greater efficacy in vaccination. Briefly, the isolated inclusion body containing 95% pure GT198 protein was repeatedly washed by sonication in PBS and sterilized by 70% ethanol. Incomplete Freund's adjuvant (IFA) was mixed with PBS at 1:1 ratio together with GT198 protein pellet, and was sonicated using a sterilized probe to produce GT198 antigen at 1 mg/ml for each subcutaneous injection at 100 μg in 100 μl (on days 0, 14, 28, 46, 63). GST was too soluble to yield inclusion body so that PBS alone was served as control. Mouse tail blood was collected at each vaccine time point to produce serum (5-10 μl). The antibody titers were measured at the end of experiment using His-tagged GT198-coated 96-well white plate (100 ng GT198 and 5 μg BSA/well), which was incubated with 200 μl of 1:1000 diluted mouse sera in duplicate wells, and detected by HRP-conjugated anti-mouse antibody with ECL detection reagents. Antibody titers were counted by a Dynex luminometer. Tumor implantations in mice brain were carried out in eight C57BL/6 mice weighing 20-22 g using luciferase-positive GL261 glioma cells (5×10^4^ in 3 μl) six weeks after the initial vaccine (n=4) or PBS (n=4) injection. For optical imaging, the living mice were intraperitoneal injected with luciferin at 150 mg/kg and optical bioluminescent imaged at weeks 2 and 3 after tumor implantation using an AMI-x optical imager.

For 4T1 breast tumor model, immunocompetent Balb/c mice were implanted with luciferase-positive 4T1 breast cancer cells (5×10^4^) two weeks after two doses of immunizations at 2 months intervals using GT198-primed matured dendritic cells. Purified soluble recombinant GST-GT198 and control GST proteins (200 μg/mouse) were used as antigens to prime dentritic cells. The living mice were intraperitoneal injected with luciferin at 150 mg/kg for bioluminescent imaging every week using an AMI-x optical imager. Photon intensity reflecting tumor growth was graphed using GraphPad Prism software.

### Statistical analysis

Statistical analyses were carried out using GraphPad Prism software. Scattergrams with means and Kaplan-Meier curves are presented using immunohistochemical staining scores in human oral cancer. *P* values in scattergrams or in bar graphs were calculated using unpaired two-tailed t test. * *P*<0.05, ** *P*<0.01, *** *P* <0.001; NS, not significant. *P* value in Kaplan-Meier curves was calculated by log-rank test. *P* value in luciferase assay was calculated by linear progression. IC50 values in MTT assay were calculated by non-linear progression sigmoidal curve fit. A *P* value of less than 0.05 is considered statistically significant.

## SUPPLEMENTARY MATERIALS FIGURES AND TABLE


