# Supplementary material for: Malignant pericytes expressing GT198 give rise to tumor cells through angiogenesis
Source: Oncotarget. 2017 May 25;8(31):51591–607. doi: 10.18632/oncotarget.18196 (PMC5584272; doi:10.18632/oncotarget.18196)
Supplement: Supplementary file 1 [file oncotarget-08-51591-s001.pdf]

Malignant pericytes expressing GT198 give rise to tumor cells through angiogenesis

SUPPLEMENTARY MATERIALS

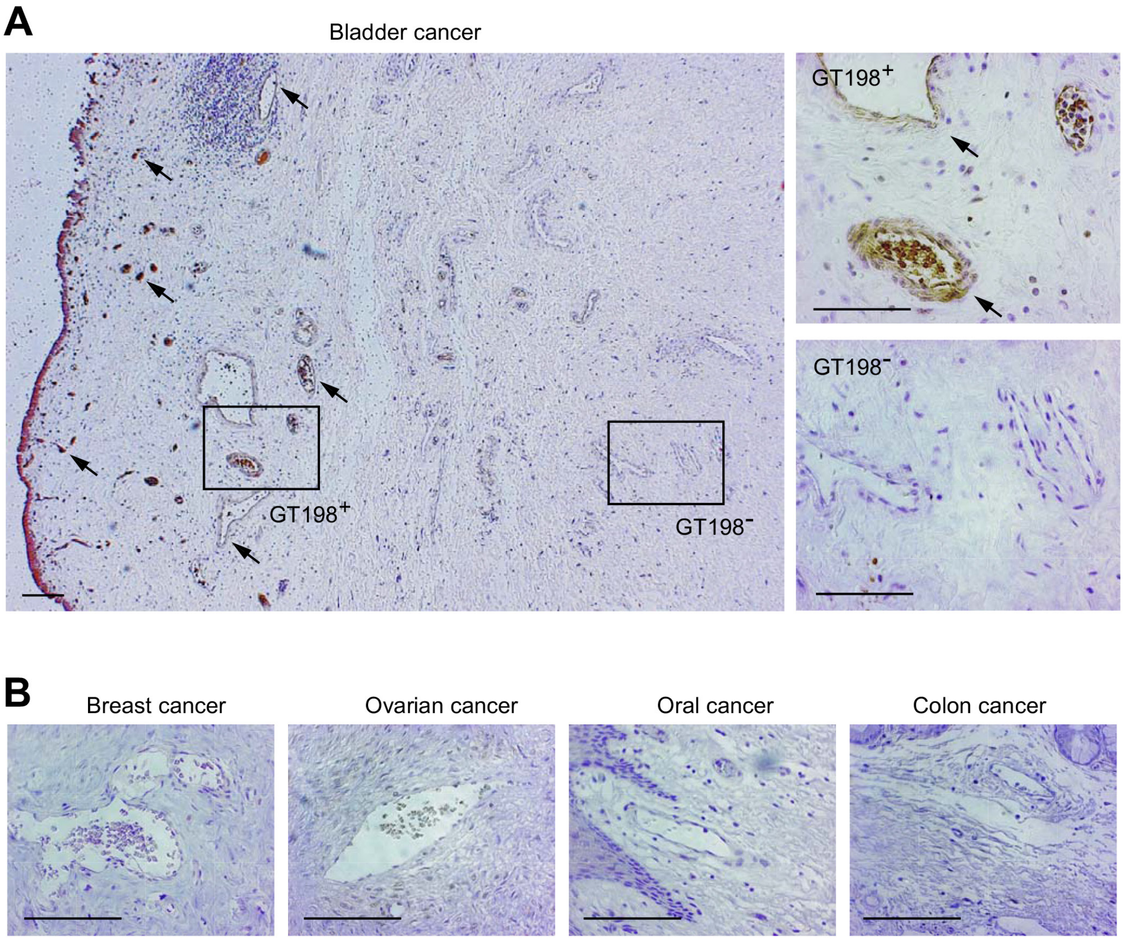

Supplementary Figure 1: GT198-negative pericytes in human tumor stroma. **(A)** Immunohistochemical staining of GT198 in a human bladder cancer with boxed areas enlarged at the right. Inflammation and angiogenesis are present in areas near epithelium (left) where small blood vessels are GT198$^+$. Arrows indicate GT198$^+$ blood vessels. In blood vessels distant from epithelium, GT198 expression is negative in pericytes. **(B)** GT198$^-$ blood vessels can be found in tumor stroma of various types of cancer where angiogenesis is not significant and pericytes are quiescent. Sections are counter-stained with hematoxylin. Scale bars = 100 μm.

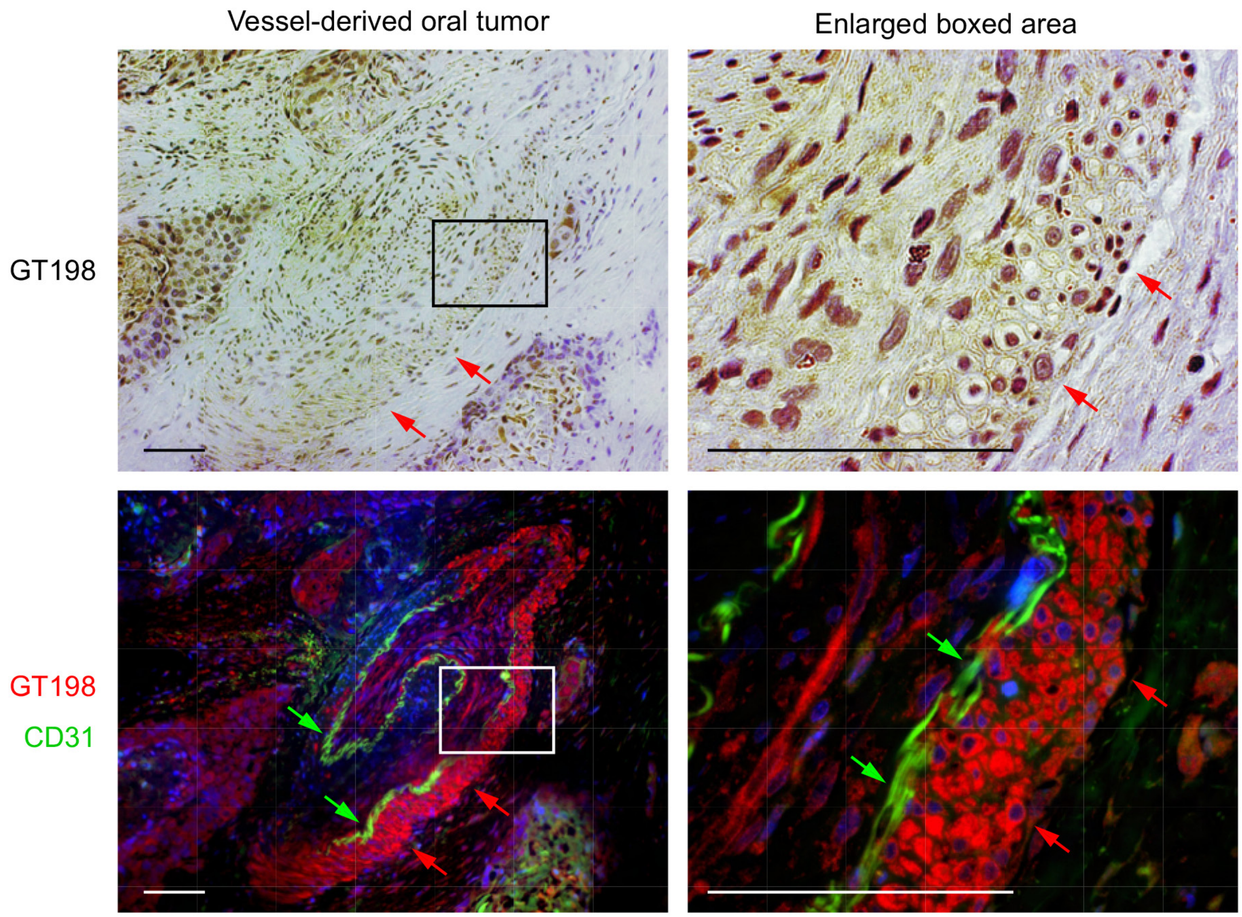

Supplementary Figure 2: CD31-positive endothelial layer in human oral tumor. Adjacent human oral tumor sections were immunohistochemical stained with GT198 (top panels), and fluorescent doubled stained with GT198 in red and CD31 in green (bottom panels). Boxed areas are enlarged at the right. Red arrows indicate GT198^{+} pericyte layer which evolves into tumor cells. Green arrows indicate CD31^{+}endothelial layer which is disintegrated and lost during tumor development. Scale bars = 100 µm.

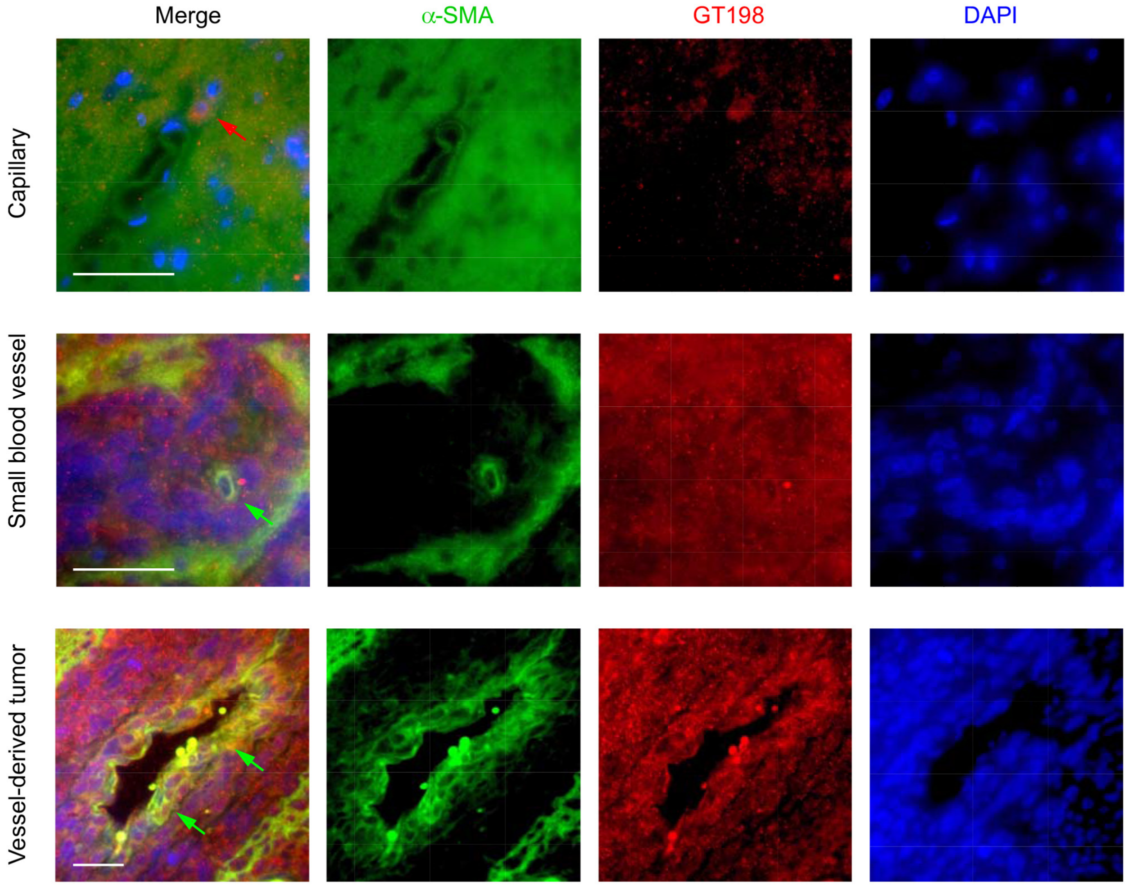

Supplementary Figure 3: GT198-positive pericytes in U-251 xenograft tumor. Fluorescent double staining of blood vessels at different sizes in U-251 xenograft tumor in rat brain. Individual channels are shown with GT198 in red, α-SMA in green, and DAPI in blue. A $GT198^{+}$ cell (red arrow) migrates to a normal capillary adjacent to tumor (top panels). A layer of α-SMA^{+} $GT198^{+}$ pericytes (green arrow) are enclosed by tumor cells when vessels become larger (middle panels). In a large vessel, α-SMA^{+} pericytes (green arrows) are $GT198^{+}$ cells which proliferate into $GT198^{+}$α-SMA^{-}tumor cells surrounding the vessel (bottom panels). Scale bars = 50 µm.

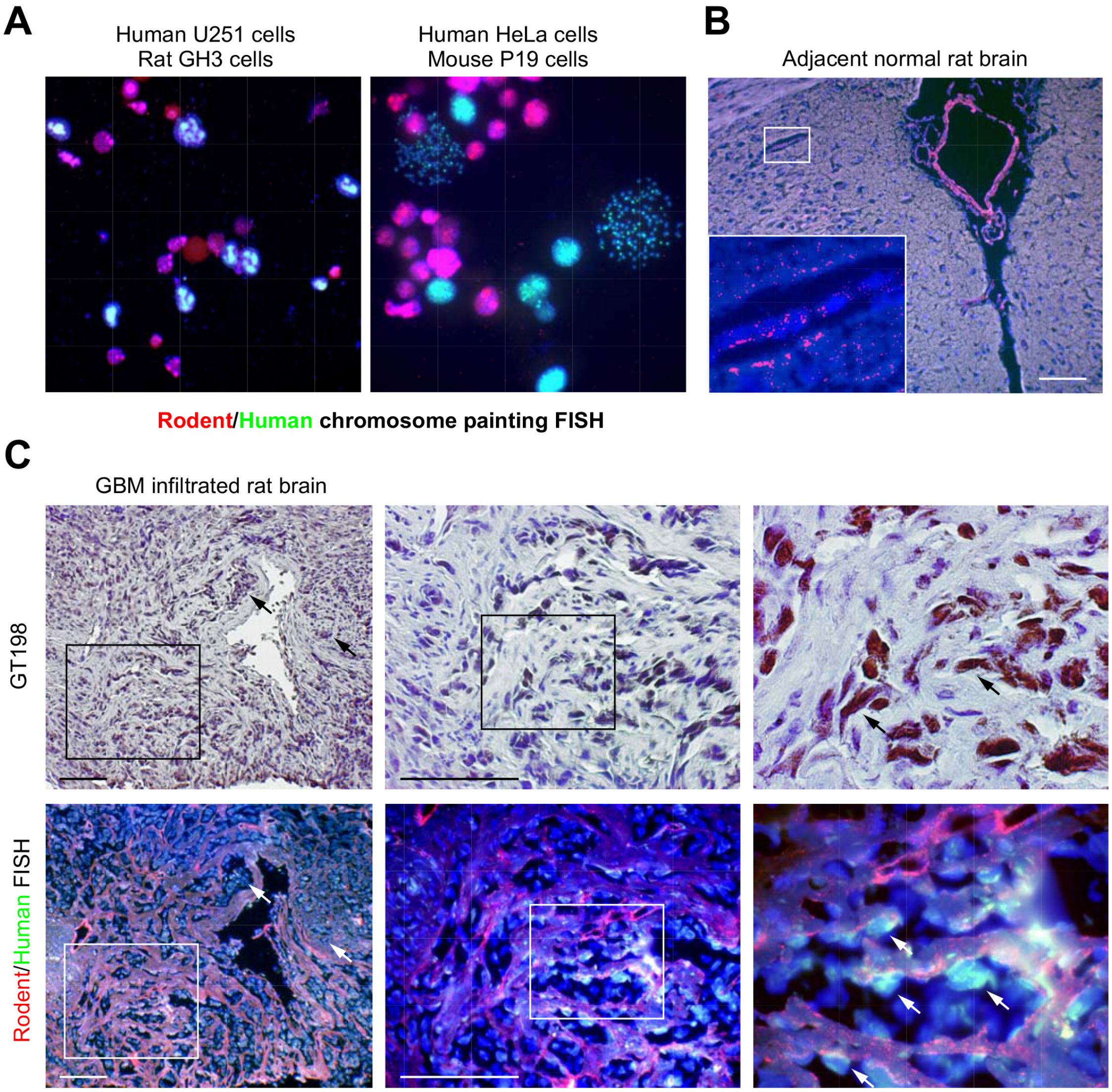

Supplementary Figure 4: FISH analysis of U-251 xenograft tumor in rat brain. (A) Validation of human chromosome painting probe in green and rodent probe in red using human U-251 and rat GH3 (left panel), or human HeLa and mouse P19 cell mixtures (right panel). **(B)** Rat brain normal tissue is stained in red. Boxed area with a blood vessel is enlarged. **(C)** FISH analysis of rat tissues in red infiltrated by human tumor cells in green. Sections are counter-stained with DAPI in blue. Arrows indicate tumor cells in green. An adjacent section was immunohistochemical stained using anti-GT198 for comparison. Sections are counter-stained with hematoxylin. Boxed areas are serial enlarged at the right. Scale bars = 100 µm.

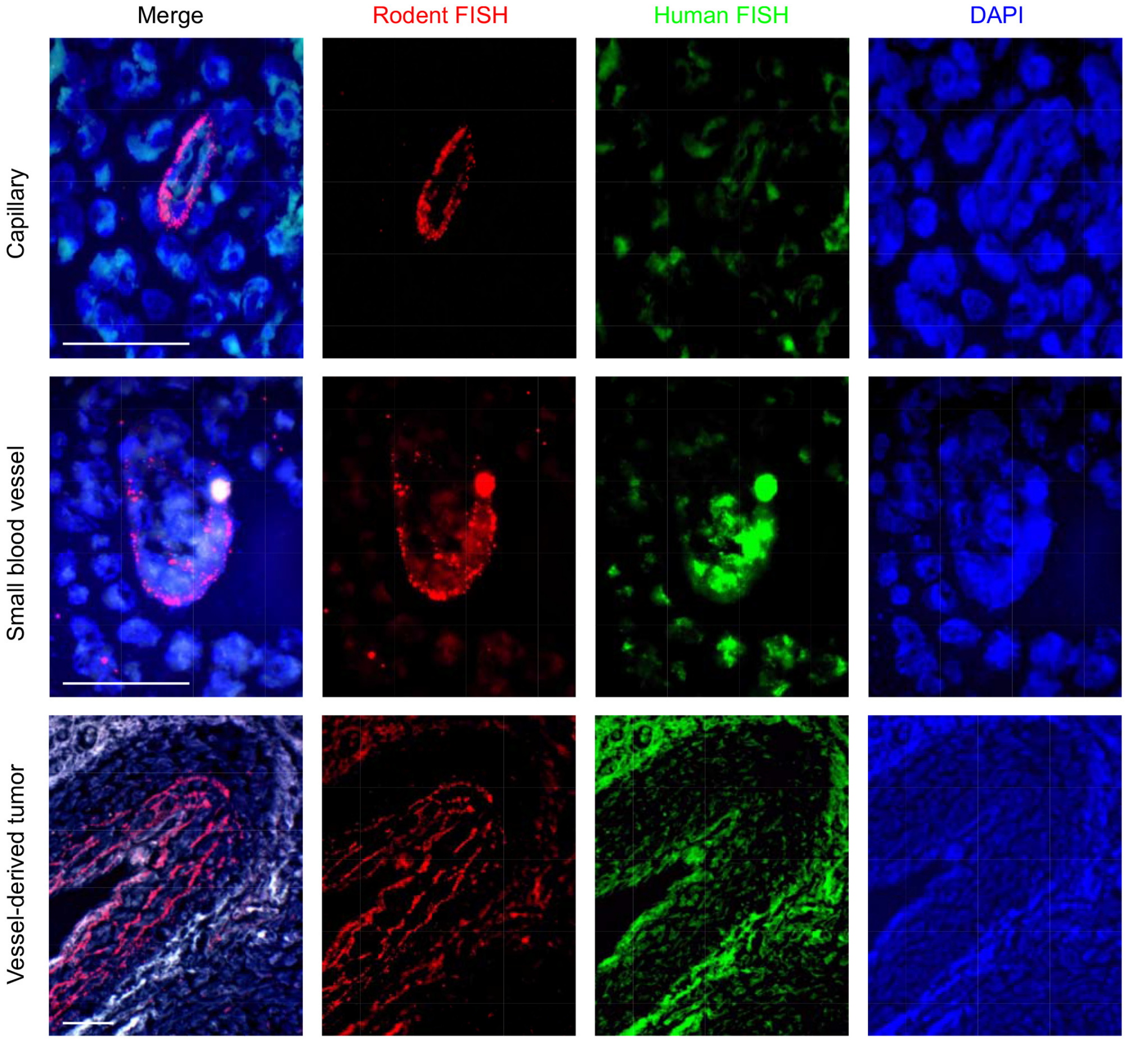

Supplementary Figure 5: Endothelial layers are disintegrated in larger tumor-derived vessels. FISH analysis of blood vessels at different sizes in U-251 xenograft tumor. Individual channels are shown with human probe in green, rodent probe in red, and DAPI in blue. A single layer of endothelium in red is found in smaller vessels and multiple layers of endothelium are found in larger vessels, which contain pericyte-derived tumor cells in thickened vessel wall. Non-specific signals are in white due to the overlay of red and green. Scale bars = 50 μm.

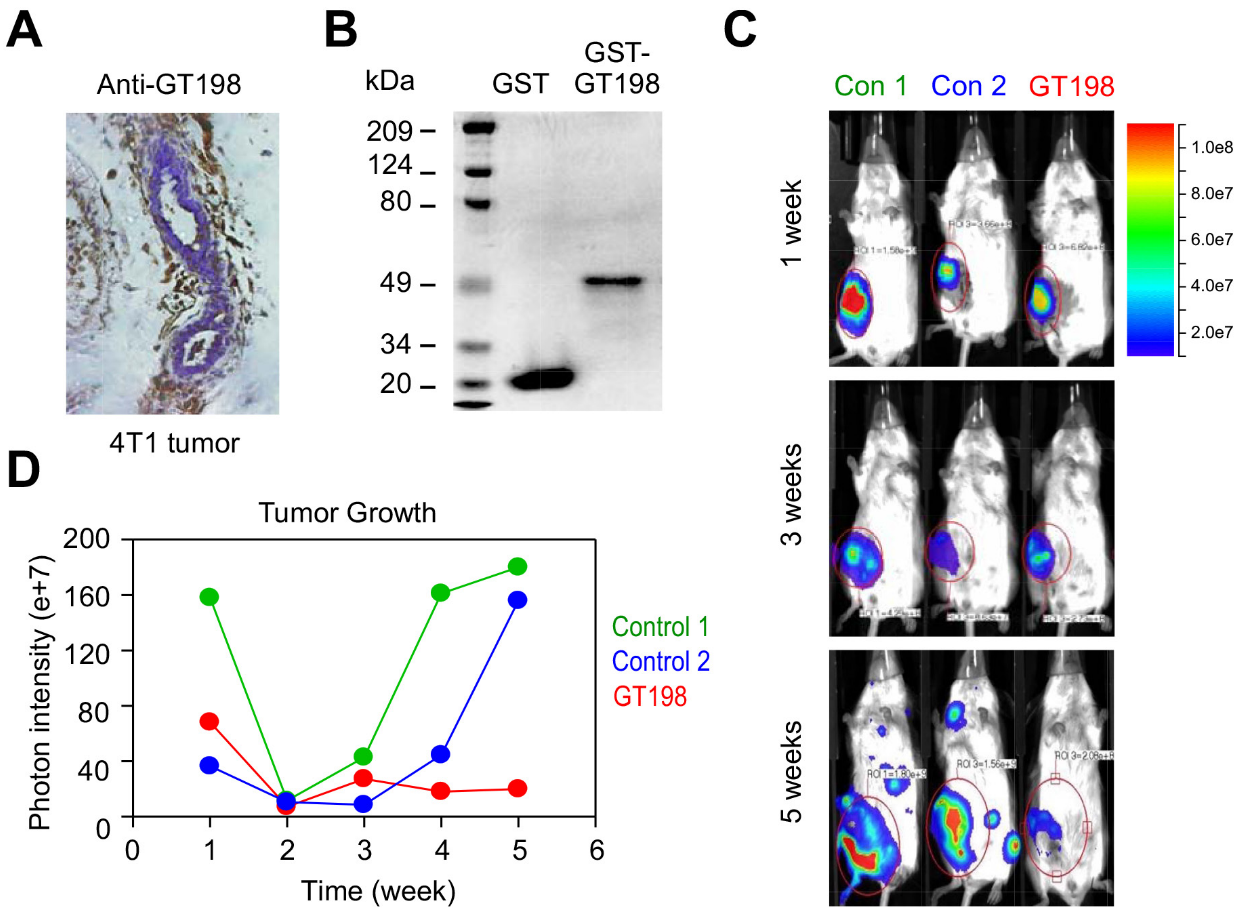

Supplementary Figure 6: Targeting GT198 inhibits tumor growth and metastasis in mouse. Immunocompetent Balb/c mice were implanted with luciferase-positive 4T1 breast cancer cells ($5x10^4$) two weeks after two doses of immunizations at 2 months intervals using GT198-primed matured dendritic cells. Luciferin was injected before bioluminescent imaging in every week. **(A)** Immunohistochemical staining of GT198 in 4T1 tumor showing the presence of positive pericytes and stromal cells. **(B)** Coomassie blue staining of purified recombinant GST and GST-GT198 proteins as antigens. **(C)** Bioluminescent imaging of live mice showing the increased tumor growth and metastasis at 5 weeks of tumor implantation in two GST-immunized control mice. In the GT198-immunized group, one mouse was censored before the end of experiment without tumor metastasis. The other mouse showed decreased tumor growth without metastasis at 5 weeks. **(D)** Tumor growth in each mouse is quantified by photon intensity.

Supplementary Table 1: Clinical features of 40 oral cancer patients

Patient ID	Gender	Age	Smoking	TNM staging	Progression-free survival month	GT198 expression score (0-7)		
						Tumor	Adjacent tissue	Lymph node
P1	M	56	S	T4AN0M0	26	1.5	-	-
P2	M	54	S	T4N0M0	22	2	-	-
P3	M	82	N	T4AN0M0	69	1	-	-
P4	M	70	S	T2N0M0	62	2	-	-
P5	F	90	N	T2N0M0	3	0	-	-
P6	F	45	S	T4AN0M0	29	3	-	-
P7	M	50	N	T4N0M0	9	2	-	-
P8	M	49	S	T4N2CM0	19	0	-	-
P9	F	89	S	T4N2BM0	13	1	-	-
P10	M	54	S	T4N1M0	13	5	-	-
P11	F	87	N	T4AN0M0	9	3	-	-
P12	M	65	S	T4AN2CM0	8	3	-	-
P13	F	79	N	T1N1MX	17	2.5	-	-
P14	M	50	S	T1N0M0	10	0	-	-
P15	M	57	N	T4AN0M0	7	0	-	-
P16	M	74	S	T4N0M0	8	0	-	-
P17	M	57	S	T4AN2BM0	14	1.5	-	-
P18	F	50	N	T1N0M0	4	2	-	-
P19	M	49	S	T1N0M0	5	3	-	-
P20	F	49	S	T1N0M0	3	5	-	-
P21	M	42	S	T4N0M0	113	2	-	-
P22	F	89	S	T1N1MX	13	1	-	-
P23	M	59	S	T1N2CM0	34	4	-	-
P24	M	79	S	T4AN0M0	4	1	-	-
P25	F	41	N	T1N0M0	3	0	-	-
P26	F	47	N	T4AN0MX	120	0	-	-
P27	M	31	S	T2N1M0	3	7	-	-
P28	F	89	S	T1N0M0	1	3	-	-
P29	M	74	S	T3N2CM0	6	2.5	-	-
P30	F	76	N	T1N0M0	62	1	-	-
P31	F	73	S	T2N0M0	6	0.5	-	-
P32	F	59	S	T1N2BMX	81	3	-	-
P33	M	44	S	T4AN2BM0	6	3	-	1
P34	M	55	S	T2N2BM0	6	2.5	-	3
P35	M	58	S	T2N2BM0	5	3	-	2
P36	F	58	N	T3N2BM0	4	1	-	2
P37	M	56	S	T4N1M0	6	4	6	0
P38	M	63	S	T2N2BM0	2	3	3	2
P39	F	46	S	T3N2CM0	5	2	3	2
P40	M	53	S	T3N2BM0	2	2	5	3

Hyphen denotes sample unavailable.
